# Cotton dust exposure and self-reported respiratory symptoms among textile factory workers in Northwest Ethiopia: a comparative cross-sectional study

**DOI:** 10.1186/s12995-018-0194-9

**Published:** 2018-04-03

**Authors:** Sintayehu Daba Wami, Daniel Haile Chercos, Awrajaw Dessie, Zemichael Gizaw, Atalay Getachew, Tesfaye Hambisa, Tadese Guadu, Dawit Getachew, Bikes Destaw

**Affiliations:** Department of Environmental and Occupational Health and Safety, Institute of Public Health, University of Gondar, Gondar, Ethiopia

**Keywords:** Respiratory symptoms, Cotton dust, Textile factory workers, Northwest Ethiopia

## Abstract

**Background:**

Cotton dust induced respiratory disorders are dramatically increased over the globe, especially the problem is serious in developing countries. Respiratory symptoms, such as cough, phlegm, wheezing, shortness of breath, chest tightness, chronic bronchitis, and byssinosis are common among workers exposed to cotton dust. However, in Ethiopia, the magnitude of the problem is not well known and information is limited about the risk factors. Therefore, this study was aimed to assess the prevalence of respiratory symptoms and associated factors.

**Methods:**

A Comparative cross-sectional study design was employed. A total of 413 (276 exposed and 137 unexposed) participants were included in the study. Stratified and simple random sampling techniques were used to select exposed and unexposed groups to cotton dust respectively. Multivariable binary logistic regression analyses was performed to identify variables associated with respiratory symptoms and adjusted odds ratio (AOR) was used to determine the strength of associations. Significance level was obtained at 95% confidence interval (CI) and *p*-value ≤0.05.

**Results:**

The prevalence of self-reported respiratory symptoms was 47.8% (95% CI: 41.3, 53.7%) and 15.3% (95% CI: 9.6, 22.3%) among exposed and control groups respectively. Sex (AOR = 2.1, 95% CI: 1.29, 3.45), service year (AOR = 2.38, 95% CI: 1.19, 4.71) and ventilation (AOR = 2.4, 95% CI: 1.17, 4.91) were factors significantly associated with respiratory symptoms. Furthermore, working department such as; ginning (AOR = 5.1, 95% CI: 2.13, 12.16), spinning (AOR = 4.96, 95% CI: 2.18, 11.29), weaving (AOR = 5.9, 95% CI: 2.46, 14.27) and blowing working departments (AOR = 5.14, 95% CI: 1.4, 18.94) were significantly associated with respiratory disorders.

**Conclusions:**

The prevalence of self-reported respiratory symptoms was higher among workers exposed to cotton dust than unexposed workers. Sex, service year, working department and work unit ventilation were predictor variables for respiratory symptoms. Thus, reducing exposure to dust, adequate ventilation and improving the hygiene of working departments are needed to reduce respiratory symptoms.

## Background

Occupational respiratory diseases are a major global public health problem that account for up to 30% of all registered work related diseases and 10–20% of deaths are caused by respiratory problems [1]. Owing to an exposure to occupational airborne particulates, an estimated 386, 000 deaths and nearly 6.6 million disability adjusted life years (DALYs) occurred among workers [2]. With respect to cotton dust exposure, chest tightness was the most common respiratory symptom (20.3%). About 14.2% of cotton processing workers were encountering byssinosis [3].

The ginning, spinning and weaving process of textile industry generate large amount of cotton dust. The dust consists of different size and type of particles, such as ground-up plant matter, fiber, bacteria, fungi, soil, pesticides, non-cotton matter, and other contaminants [4, 5]. Exposure to the cotton dust led to respiratory problems, such as cough, phlegm, wheezing, shortness of breath, chest tightness, chronic bronchitis, and byssinosis [6–8]. Exposure to cotton dust has also profound effect on pulmonary function [8]. Byssinosis is a chronic respiratory disease that is seen among workers exposed to cotton dust [9, 10]. The type and concentration of dust, duration of exposure and genetic factors are interplaying the diseases of the respiratory system induced by occupational dusts [11, 12]. Moreover, working in the department where there is higher exposure of cotton dust such as spinning and weaving and being aged were found to be the risk factors for respiratory problems related to cotton dust [10].

Respiratory problems related to coton dust start to decline in developed countries as a result of stringent measures taken by the employers and workers. However, the problems are quite neglected in developing countries [13, 14] and there is lack of health information.

Textile industry is one of the major manufacturing industry, which is established across the developed and developing countries including Africa [15]. In developing countries, notably in Africa, the cotton industry occupies an important place. The cotton sector is expanding considering the size of cotton production and the number of people employed in this sector [10]. In Ethiopia, textile industry or cotton sector is the main economic motor that attracts numerous work forces [16]. However, the workers are at risk of cotton dust related respiratory problems. But the degree of the problem is not well known and there is a scarcity of data showing these kinds of health issues and its risk factors in Ethiopia.

Therefore, this study was conducted to assess cotton dust exposure, self-reported respiratory symptoms and associated factors among textile factory workers.

## Methods

### Study design and study population

A Comparative cross-sectional study design was employed in northwest Ethiopia textile factory.

The source population of this study was all workers in northwest Ethiopia textile factories and those who were worked in ginning, spinning, weaving and blowing departments. Those workers who worked for more than 1 year were included in the study. However, workers with previous exposure to other occupational dust such as silica, coal dust and who have history of smoking were excluded. Moreover, workers who had history of asthma or chronic obstructive pulmonary disease (COPD) before joining to the work were also excluded from this study.

Regarding the activities carried out in working unit, Ginning section involves the process of separating of the cotton fibers from the seed for the conversion of the cotton into a continuous thread. Cotton ginning consists simple operations, which was done mechanically. During the ginning process, dust fibers and lint are generated which can be inhaled by workers.

Blowing section is the initial stage in spinning process. Blow department is consisting of different machines in which the supplied compressed bales are opened, cleaned, dust removed, mixing or blending performed for making uniform lap of definite length. It is sued in succession to open and clean the cotton fiber according to the required amount of degree.

The Spinning department uses machines in order to produce cotton into threads of required size from the locks of cotton.

The Weaving department comprises different activities such as winding, warping, gluing and weaving. Then, cloth is obtained from the chain threads placed vertically and the weft threads placed horizontally. Cloths are afterwards stocked in the warehouse.

The source population for unexposed group were both the general administration staff members of the textile factories and external workers in the informal sectors located in the surroundings (estimated to be 200–500 meter far from technical department of textile factory) having at least 1 year of job activities. Those who have history of smoking, asthma or COPD were excluded from unexposed group.

### Sample size determination

The sample size was determined using double population proportion formula using EpiInfo software considering the following assumptions: proportion of respiratory symptoms among exposed group (36.9%) [10], proportion of respiratory symptoms among unexposed group (21.2%) [10], 95% confidence interval, 80% power, margin of error (5%), 2:1 ratio of exposed to unexposed groups. A total of 413 study participants, 276 exposed and 137 unexposed groups were included in this study.

### Sampling procedures

Study subjects from the exposed groups were selected using stratified sampling technique, assuming that workers in different departments would exhibit different level of exposure to cotton dust. Study subjects were allocated to each stratum proportionally and selected by simple random sampling. Whereas, the unexposed groups were selected by simple random sampling technique using their salary payment roaster sheet, which was obtained from their respective offices.

### Measurment of variables

Respiratory symptoms, the primary outcome variable of the study was determined by the presence of one or more respiratory symptoms such as, cough, phlegm, wheezing, dyspnea, chest pain and breathlessness among workers.

Ventilation condition, the ventilation condition of the working units was reported as adequate if the working unit furnished with functional mechanical ventilation system (ventilator, Local exhaust ventilation system) and natural ventilation systems (doors, windows and any other openings). Lack of obstruction of air flow in the unit also considered and if the data collector senses sufficient air circulation in the working unit; fair if there is functional mechanical ventilation system and natural systems, but obstructed air flow due to poor design of the working units; and inadequate if the unit lacks functional mechanical and natural ventilation system, and if the air flow obstructed by adjacent buildings and poor layout of the unit.

### Data collection procedures

The data were collected by using a modified questionnaire of medical research council (MRC) of Great Britain and work place observation checklists. The questionnaire consisted of three parts, like socio-demographic, environmental and behavioral factors, and respiratory symptoms. Face-to-face interview and observation of the working units were performed to collect data. Prior to the data collection, training was given for data collectors and supervisors for 3 days on procedures, techniques and ways of collecting the data. Clear introduction explaining the purpose and objective of the study was provided to the respondents on the first page of the questionnaire before data collection. In addition continuous and strict supervision and on spot checking was carried out during the data collection process.

### Data processing and analysis

The data were checked, coded and entered in to epidemiological information package (EPi-info) version 7.2.0.1 and exported to statistical package for social sciences (SPSS) version 20 for further analysis. For most variables, data were presented as frequencies and percentages. Univariate logistic regression analysis was performed primarily to select variables for the final model on the basis of *p*-value < 0.2. Multivariable binary logistic regressions analysis was employed to control the possible effect of confounders and finally the variables which had significant association were identified on the basis of AOR with 95% CI and *p*-value ≤ 0.05.

## Results

### Demographic and socio-economic characteristics of the study participants

Of the total 413 questionnaires (276 exposed and 137 unexposed) distributed, 401 (270 exposed and 131 unexposed) completed and valid questionnaires were analyzed, which gives a response rate of 97.1%.

Two third, 169 (62.6%) of exposed and half, 66 (50.4%) of unexposed participants were males. The mean age (± SD) of the respondents among exposed and unexposed was 28.24 (± 7.58) and 29.79 years (± 7.4) respectively (Table 1).

**Table 1:** Demographic and socio-economic characteristics of textile factory workers, northwest Ethiopia, 2017 (*N* = 413)

Variables	Exposed frequency (%)	Unexposed frequency (%)
Sex		
Female	101 (37.4%)	65 (49.6%)
Male	169 (62.6%)	66 (50.4%)
Age(years)		
≤ 29	199 (73.7%)	74 (56.5%)
30–39	43 (15.9%)	42 (32.1%)
≥ 40	28 (10.4%)	15 (11.5%)
Mean ± SD	28.24 ± 7.58	29.79 ± 7.4
Marital status		
Single	158 (58.5%)	54 (41.2%)
Married	109 (40.4%)	76 (58%)
Divorced/Widowed	3 (1.1%)	1 (0.8%)
Years of service in current industry (years)		
1–2	56 (20.7%)	14 (10.7%)
2–5	118 (43.7%)	83 (63.4%)
≥ 6	96 (35.6%)	34 (26.0%)
Monthly salary		
≤ 1500	163 (60.4%)	37 (28.2%)
1501–2500	87 (32.2%)	46 (35.1%)
≥ 2501	20 (7.4%)	48 (36.6%)

### Workplace condition

The overall conditions of employees and the working environment were observed to see workers exposure to cotton dust. Accordingly, poor indoor air quality at working environment was observed. Working environments were characterized with excessive dust and there was no a documented functioning housekeeping program. Moreover, most of the data collectors were experienced sudden sneezing upon entering the working unit in the textile industries and it was observed that worker’s eye brows, hair, nostrils and cloths were covered with dust particle.

Although, natural ventilation systems (doors, windows and other opening) were present, the air flow in different working units was obstructed due to poor design and layout of the working units. The working units lacked functional mechanical ventilation system. In addition, working units were poorly illuminated. We observed that all the working units had no warning signs and safety instruction procedures. Safety procedures were not posted indicating whether employees cotton dust exposure is kept at the accepted levels. None of the plants practiced wet mopping to minimize cotton dust exposure. We also found that all of the workers did not use respirator, face shields, and other PPE to minimize cotton dust exposure.

### Respiratory symptoms

This study revealed that the prevalence of self-reported cotton dust induced respiratory symptoms were 47.8% (95% CI: 41.3, 53.7%) among exposed and 15.3% (95% CI: 9.6, 22.3%) among unexposed groups. Cough (28.1%), phlegm (19.6%), chest tightness (30%) and dyspnea (21.11%) were the commonest respiratory symptoms reported by the exposed groups. Significanct differences of respiratory symptoms were observed between exposed and unexposed participants and there were more signs of respiratory tract irritations among exposed workers than the unexposed participants (*P*-value ≤0.05) (Table 2).

**Table 2:** Prevalence of respiratory symptoms among textile factory workers, northwest Ethiopia, 2017 (*N* = 413)

Respiratory symptoms	Exposed *n* (%)	Unexposed *n* (%)	*P*-value
Cough	76 (28.1%)	13 (9.9%)	≤ 0.001
Phlegm	53 (19.6%)	4 (3.1%)	≤ 0.001
Chest tightness	81 (30%)	11 (8.4%)	≤ 0.001
Dyspnea	57 (27.1%)	6 (4.6%)	≤ 0.001

### Association of cotton dust exposure with respiratory symptoms

High prevalence of respiratory symptoms was reported among exposed participants than unexposed to cotton dust (*P*-value ≤0.001). Twenty (15.3%) of unexposed workers and 129 (47.8%) workers exposed to cotton dust experienced one or more respiratory symptoms.

### Factors associated with respiratory symptoms

In the bivariate binary logistic regression sex, monthly salary, ventilation of the working unit, working departments and PPE use had *p*-value < 0.2. However, only sex, service year and ventilation of the working units had statistically significant association with respiratory symptoms in the multivariable binary logistic regression analysis (Table 3).

**Table 3:** Multi variable analysis of factors associated with Factors associated with respiratory symptoms among textile factory workers, northwest Ethiopia, 2017 (*N* = 413)

Variables	Respiratory symptoms		COR (95% CI)	AOR (95% CI)
	No	Yes		
Sex				
Female	123	43	1.00	1.00
Male	129	106	2.35 (1.53, 3.62)	2.1(1.29, 3.45)^*^
Age(years)				
≤ 29	170	103	1.00	1.00
30–39	56	29	0.86 (0.51, 1.43)	1.6 (0.81, 3.14)
≥ 40	26	17	1.1 (0.56, 2.1)	1.63(0.67, 3.98)
Marital status				
Single	124	88	1.00	1.00
Married	125	60	0.68(0.45, 1.02)	0.8 (0.48, 1.39)
Divorced/Widowed	3	1	0.47(0.05, 4.6)	0.45(0.04, 5.18)
Educational level				
Primary school	20	12	1.00	1.00
Secondary school	43	44	1.7 (0.74, 3.91)	1.66(0.67, 4.1)
Diploma and above	189	93	0.82 (0.38, 1.75)	1.29(0.51, 3.27)
Monthly salary				
≤ 1500	123	77	1.00	1.00
1501–2500	77	56	1.16 (0.74, 1.82)	1.3(0.79, 2.26)
≥ 2501	52	16	0.49 (0.26, 0.92)	0.87(0.38, 1.98)
Years of service in current industry (years)				
1–2	49	21	1.00	1.00
2–5	121	80	1.5 (0.86, 2.77)	2.38 (1.19, 4.71)^*^
≥ 6	82	48	1.4 (0.73, 2.55)	1.51(0.68, 3.36)
Working department				
Ginning	41	41	5.5 (2.92, 10.56)	5.1(2.13, 12.16)^**^
Spinning	50	40	4.4 (2.36, 8.35)	4.96 (2.18. 11.29)^**^
Weaving	43	41	5.3 (2. 8, 10.04)	5.9(2.46, 14.27)^**^
Blowing	7	7	5.6 (1.76, 17.5)	5.14(1.4, 18.94)^*^
Administration/informal sector	111	20	1.00	1.00
Ventilation of the working unit				
Adequate	58	16	1.00	1.00
Fair	127	70	1.99 (1.07, 3.74)	2.1(1.07, 4.19)^*^
Inadequate	67	63	3.41(1.78, 6.54)	2.4 (1.17, 4.91)^*^
PPE use				
No	148	61	1.00	1.00
Yes	104	88	2.05 (1.36, 3.1)	099(0.59, 1.66)

Note: ^* =^ *P*-value ≤0.05, ** = *p*-value ≤0.001

According to this study male respondents had 2.1 times higher odds of developing respiratory symptoms when compared to female participants (AOR = 2.1, 95% CI: 1.29, 3.45). Employees with two to 5 years of service year had 2.38 times higher odds of having respiratory symptoms than employees with less than 2 years of work experience (AOR = 2.38, 95% CI: 1.19, 4.71).

Moreover, working department was significantly associated with respiratory symptoms. The prevalence of respiratory symptoms was higher in ginning (50%), blowing (50%), weaving (48.8%) and spinning departments (44.4%). Respondents in ginning and spinning working departments had 5.1 and 4.96 times higher odds of developing respiratory symptoms respectively than workers in administrative units and other sectors (AOR = 5.1, 95% CI: 2.13, 12.16, and AOR = 4.96, 95% CI: 2.18, 11.29). Furthermore, workers in weaving and blowing working departments had 5.9 and 5.14 times higher odds of having respiratory symptoms respectively compared to workers in administrative units and other sectors (AOR = 5.9, 95% CI: 2.46, 14.27, and AOR = 5.14, 95% CI: 1.4, 18.94).

Workers in inadequatly ventilated working units had 2.4 times higher odds of developing respiratory symptoms compared to their counter parts (AOR = 2.4, 95% CI: 1.17, 4.91).

## Discussion

In the current study, the prevalence of self reported respiratory symptoms was higher among participants exposed to cotton dust (47.8%) than unexposed respondents (15.3%). The result of this study is in line with the reports of other similar studies outside Africa, like India [17], Pakistan [18] and China [19]. This might be due to the fact that exposure to dust from cotton during weaving, spinning, ginning and packaging are higher among textile workers. Hence, occupational exposure to cotton dust has been linked with respiratory disorders.

According to this study, male respondents had higher odds of developing respiratory symptoms. This finding is supported by study done in Shanghai and Lancashire cotton industries indicating that male textile workers are at higher risk of having respiratory symptoms than female textile workers [20, 21]. The difference in respiratory symptoms might be explained due to the fact that male workers have longer service year in this study and have higher cumulative dust exposure. Other possible reason is, female workers are usually assigned in less hazardous department in cotton industries [20, 21]. However, this finding is contrary to studies done in Greek [22], Denmark [23] and China [24].

Employees who have longer service years had higher odds of having respiratory symptoms. This finding was similar with studies done in Egypt [25] and India [24, 26]. This result shows, the longer the duration of exposure to cotton dust the higher the presence of respiratory symptoms. This can be justified as more experienced workers had prolonged exposure to cotton dust in cotton industries they are at greatest risk for developing respiratory symptoms [22, 23].

Moreover, working department was significantly associated factor with respiratory symptoms. The prevalence of respiratory symptoms was higher in ginning, weaving, spinning and blowing departments. This finding is similar with studies done in Pakistan in 2015 [27] and in 2017 [28] and in Egypt [25]. This is due to the fact that worker in higher cotton dust exposure work environment such as blowing are more likely to develop respiratory symptoms than those workers with in administrative areas where there is less cotton dust exposure [18]. Furthermore, cotton dust is high in these working units because of the work nature and poor environmental conditions [29].

Ventilation of working units was statistically associated with respiratory symptoms. Higher odds of developing respiratory symptoms were observed among workers who worked in inadequately ventilated working units. This result is not surprising because textile industries release high level of dust in working environment [30, 31] and various studies indicated that lack of ventilation in cotton industry working environment as a major factor of developing respiratory symptoms among cotton industry workers [32]. Moreover, their effects have a tendency to be more pronounced in the case of poor ventilation [33].

Lack of the Pulmonary Function Test and measurement of cotton dust concentration to strengthen the self-reported symptoms were the limitations of this study. In addition, the possibility of healthy workers effect could not be ruled out. But through observation of overall conditions of employees and working environment, excluding workers with less than 1 year of work experience and by honestly explaining the objective and significances of the study we tried to minimize the effect.

## Conclusion

The prevalence of self-reported respiratory symptoms were higher among participants exposed to cotton dust than unexposed respondents and there were more signs of respiratory tract irritations among workers exposed to to cotton dust. Sex, years of service, working department and work unit ventilation are risk factors for the presence of respiratory symptoms. Thus, reducing exposure to dusts, adequate ventilation and improving hygiene of the working department are needed to reduce respiratory problems.

## Abbreviations

AOR: Adjusted odd ratio
CI: Confidence interval
COPD: Chronic obstructive pulmonary disease
DALYS: Disability-adjusted life years
ILO: International Labor Organization
IRB: Institutional review board
PPE: Personal protective equipment
SPSS: Statistical package for social sciences
